# IGF2‐derived miR‐483‐3p associated with Hirschsprung's disease by targeting FHL1

**DOI:** 10.1111/jcmm.13756

**Published:** 2018-08-02

**Authors:** Zhengke Zhi, Hairong Zhu, Xiaofeng Lv, Changgui Lu, Yang Li, Feng Wu, Lingling Zhou, Hongxing Li, Weibing Tang

**Affiliations:** ^1^ Department of Pediatric Surgery Children's Hospital of Nanjing Medical University Nanjing China; ^2^ State Key Laboratory of Reproductive Medicine Institute of Toxicology School of Public Health Nanjing Medical University Nanjing China; ^3^ Department of Gastroenterology Zhongshan Hospital of Fudan University Shanghai China

**Keywords:** four and a half LIM domains 1, gene regulation, Hirschsprung's disease, insulin‐like growth factor 2, miR‐483‐3p

## Abstract

HSCR (Hirschsprung's disease) is a serious congenital defect, and the aetiology of it remains unclear. Many studies have highlighted the significant roles of intronic miRNAs and their host genes in various disease, few was mentioned in HSCR although. In this study, miR‐483‐3p along with its host gene IGF2 (Insulin‐like growth factor 2) was found down‐regulated in 60 HSCR aganglionic colon tissues compared with 60 normal controls. FHL1 (Four and a half LIM domains 1) was determined as a target gene of miR‐483‐3p via dual‐luciferase reporter assay, and its expression was at a higher level in HSCR tissues. Here, we study cell migration and proliferation in human 293T and SH‐SY5Y cell lines by performing Transwell and CCK8 assays. In conclusion, the knockdown of miR‐483‐3p and IGF2 both suppressed cell migration and proliferation, while the loss of FHL1 leads to opposite outcome. Furthermore, miR‐483‐3p mimics could rescue the negative effects on cell proliferation and migration caused by silencing IGF2, while the FHL1 siRNA may inverse the function of miR‐483‐3p inhibitor. This study revealed that miR‐483‐3p derived from IGF2 was associated with Hirschsprung's disease by targeting FHL1 and may provide a new pathway to understand the aetiology of HSCR.

## 1 INTRODUCTION

To form a complete functional enteric nervous system (ENS), the normal migration, proliferation and survival of neural crest cells within the gastrointestinal trac were required from the fifth to 12th week of embryonic stage. Any event that can disrupt these mentioned processes may cause Hirschsprung's disease (HSCR),^1^ which is regarded as a serious congenital disease with postponement of meconium, abdominal distention and so on.^2^, ^3^ In Asia, the morbidity rat of HSCR is up to 2.8:10 000 that is much higher than other continents, such as Europe; and the gender ratio is around 4:1 (male: female) worldwide.^4^ As well‐known, the specific pathogeny of HSCR is not yet clear, but some studies have proved that the occurrence and development of HSCR is the consequence of the coordination of genetic and environmental factors.^4^, ^5^ On account of it is a congenital defect, genetic factors may play a more important role in its pathogeny; thus, it is very necessary to explore the mechanisms of HSCR from the gene aspect.

MicroRNAs (miRNAs) are endogenous small non‐coding RNAs with the length of about 22nt which could enhance mRNA degradation or translational repression via binding to 3′‐UTR regions of target mRNAs,^6^, ^7^ and the diverse roles of miRNAs in malignant tumours have been studied well. In multiple myeloma, miR‐221 could cause the drug resistance by abating the expression level of its target gene PUMA.^8^ In gallbladder cancer, miR‐130a mediated the oncogenic activity of HOTAIR because of HOTAIR harbours a miRNA‐130a binding site so HOTAIR could negatively regulated it.^9^ However, the dysregulation of miRNAs in HSCR^10^, ^11^ has not been stated thoroughly to date. Benefiting from the microarray of miRNAs which was performed in our previous study,^12^ a great deal of up‐ or down‐regulated miRNAs were sought out in HSCR samples compared with the normal ones. Among them, miR‐483‐3p gained our attention because it was found down‐regulated in HSCR and transcribed from its host gene IGF2. Depending on these findings, it impelled us to further demonstrate what function miR‐483‐3p would exert in HSCR.

IGF2 (Insulin‐like growth factor 2) is a common poly‐peptide produced majorly by liver and it is well‐known as an important regulator of bodyweight and lipid metabolism.^13^ However, more and more studies have reported that IGF2 can also influence the cell survival, growth and proliferation,^14^ and the aberrant IGF2 expression is associated with the characteristic of many cancers just like high mortality of breast tumour^15^ and poor prognosis of oesophageal cancer.^16^ What's more, IGF2 plays a great role during the growth of embryonic period.^17^ HSCR is a congenital disease occurred in the embryonic period as well. Consistent with miR‐483‐3p, IGF2 was also down‐regulated in HSCR tested via qRT‐PCR and it enhanced us to reveal whether the dysregulation of IGF2 has some influences on the occurrence or development of congenital megacolon or not.

In general, 33 per cent of mammalian miRNAs appear to reside within the introns of protein‐coding genes now,termed as intronic miRNAs.^18^ Most of the intronic miRNAs are transcribed from the same initial transcripts and have a tight relationship with the expression of their host genes by the forecast of bioinformatics analyses.^19^ In addition, some miRNAs are surprisingly able to regulate their host genes in turn, for example, miR‐301 can cut down the expression of its host gene ska2 by miR‐301 inhibitor.^20^ The same situation occurs between IGF2 and miR‐483‐5p (the other IGF2 intronic miRNA) as well.^21^ In addition, some studies proved that they may cooperate to exert function, just like miR‐33 and its host gene SREBP which could work together to control Cholesterol Homeostasis.^22^ Relying on the unique regulatory relationship between miR‐483‐3p and IGF2, it made us to explore the potential functional mechanism of them in HSCR.

According to the website prediction, FHL1 (Four and a half LIM domains 1), a potential target gene of miR‐483‐3p got our great attention which was up‐regulated in HSCR confirmed by qRT‐PCR. FHL1 was reported to take part in skeletal and cardiac muscle growth initially.^23^, ^24^ Except the role of it in skeletal and cardiac muscle growth, FHL1 has also been regarded as a tumour suppressor in some cancers recently, like in lung cancer^25^ and gastric cancer^26^ because of its effects on cell migration and proliferation. However, the role of it in HSCR is still poorly understood.

Here we focused on miR‐483‐3p that resides in IGF2 and explored the role of miR‐483‐3p in the progress of HSCR. Finally, this study indicated that the down‐regulation of intronic miR‐483‐3p was involved in HSCR by controlling the expression of FHL1.

## 2 MATERIALS AND METHODS

### 2.1 Ethics statement and samples collection

This research has got approval from the Institutional Ethics Committee of Nanjing Medical University, and all activities involved in this study were carried out in accordance with government policies and the Helsinki Declaration. In total, 120 human colon tissues (collected from October 2009 to October 2014 after surgery) were obtained from Nanjing Children's Hospital Affiliated to Nanjing Medical University including 60 from HSCR patients determined by a pathological diagnosis and 60 matched controls. We stored samples at −80°C immediately after operation, and all subjects provided written informed consent for us.

### 2.2 RNA extraction and qRT‐PCR

Trizol reagent (Invitrogen Life Technologies Co, CA, USA) was used for isolating total RNA from tissues and cells according to standard instructions. To evaluate the expression levels of mRNA and miRNA in tissue samples, the qRT‐PCR was performed using the ABI 7900HT (Applied Biosystems, CA, USA) and GAPDH was used as an endogenous control. In addition, the expression of miR‐483‐3p was determined by TaqMan^®^ MicroRNA Assays (Applied Biosystems) with a normalized control. The sequence of primers is as follows: IGF2(F:TACTTCAGCAGGCCCGCAAG,R: GGTGACGTTTGGCCTCCCTG),FHL1(F:CTGAAGTGCTTTGACAAGTTC,R:GTGCCAGTAGCGATTCTTAT), GAPDH(F:TGTTCGTCATGGGTGTGAAC,R:ATGGCATGGACTGTGGTCAT), hsa‐miR‐483‐3p(F:ACACTCCAGCTGGGTCACTCCTCTCCTCC,R:CTCAACTGGTGTCGTGGAGTCGGCAATTCAGTTGAG AAGACGGG).

### 2.3 Western blot

Using a RIPA buffer (Beyotime, Nantong, China), the tissue samples and cells were lysed to extract the proteins. In the next step, protein concentration was examined with BCA method (Beyotime). Equal amount of the proteins were loaded in 12% SDS‐PAGE for electrophoretic separation and then transferred to PVDF membrane (Roche Germany). Later, the primary antibodies were applied for incubation at 4°C overnight after the PVDF membranes containing proteins were blocked in 5% skimmed milk for 60 minutes.1 × TBST buffer was used to wash the membranes (three times); subsequently, the membranes were incubated with secondary antibodies (Beyotime) for about 60 minutes at room temperature.

### 2.4 Cell culture and cell transfection

Human 293T and SH‐SY5Y cell lines were purchased from American Type Culture Collection (ATCC, Manassas, VA, USA). Bothe cell lines were cultured under the condition of 37% and 5%CO2 in DMEM (Hyclone, UT, USA), supplemented with 10%FBS, 100 U/mL penicillin and 100 μg/mL streptomycin. Transfection experiments of synthetic mimics or inhibitor of hsa‐miR‐483‐3p (hsa‐mir‐483‐3p mimics UCACUCCUCUCCUCCCGUCUU, GACGGGAGGAGAGGAGUGAUU; hsa‐mir‐483‐3p inhibitor AAGACGGGAGGAGAGGAGUGA), siRNAs of FHL1/IGF2 and negative controls (GenePharma, Shanghai, China) was performed using Lipofectamine 2000 Reagent (Invitrogen, CA, USA).

### 2.5 Cell transwell assays

Transwell assay was applied to measure the ability of cell migration after transfection. Around 100 uL (1 × 10^6 cells/mL) cells mixed with medium without serum was planted to the upper chamber and meanwhile 600 uL medium containing 10%FBS in the lower chamber. Twenty‐four to 48 hours later, migrated cells were stained with crystal violet (Beyotime) and counted with Image‐pro Plus 6.0 under 40 × magnification (five views per well). All experiments were repeated three times independently.

### 2.6 Cell proliferation analysis

Cells were added in 96‐well plates, and then, CCK8 assay (Beyotime) was carried out to assess the ability of cell growth through measuring the absorbance at the wavelength of 450 nm by the TECAN infinite M200 Multimode microplate reader (Tecan, Mechelen, Belgium). All data were gained from three independent experiments.

### 2.7 Flow cytometry analysis

Cells were harvested after transfection for the detection of cell cycle by BD Biasciences FACS Calibur Flow Cytometry (BD Biasciences, NJ, USA). What's more, Annexin V‐FITC/Propidium Iodide Kit (KeyGen Biotech, Nanjing, China) was used for analysing cell apoptosis and data were analysed by FlowJo V7 software V7 software (Tree Star, OR, USA). Triplicate separated experiments have been performed in this study.

### 2.8 Luciferase assay

The 3′‐UTR sequences of FHL1 containing the miR‐483‐3p binding fragments and mutated 3′‐UTR sequences were cloned into the KpnI and SacI sites of pGL3 promoter vector (Genscript, Nanjing, China) for performing Dual‐luciferase reporter assay (Promega, Madison, WI, USA). On the basis of manufacturers’ protocols, after transfected with negative control, miR‐483‐3p mimics, pGL3‐FHL1 and pGL3‐FHL1‐mut for about 48 hours, cells were harvested for measuring the fire fly and renilla luciferase activities.

### 2.9 Statistical analysis

STATA9.2 (Stata Corp, TX, USA) was used for statistical analysis, and results were presented by Graph PAD prism software. Furthermore, all data of tissue and cell samples were analysed, respectively, with Wilcoxon rank‐sum test and presented as mean±SEM from three independent experiments by double‐sided Student's *t* test. Once *P* < .05, the results were considered statistically significant.

## 3 RESULTS

### 3.1 Clinical information analysis

In total, 120 human colon tissue specimens were applied to this study, including 60 tissues without ganglion cells from HSCR patients and 60 normal colon tissues from other patients. Clinical information of these 120 subjects mainly comprising age, gender rate (Male/Female) and bodyweight was calculated for further analysis. Among them, the age (3.80 ± 0.24 and 3.70 ± 0.26 months old) and bodyweight (5.70 ± 0.23 and 5.59 ± 0.27 kg) have no statistical significance between the HSCR and normal subjects. Besides, gender rate (Male/Female) was 49/11 in HSCR group while that of controls was 47/13 and matched the characteristic of this disease in human beings.

### 3.2 Down‐regulation of miR‐483‐3p in HSCR

**Figure 1:**
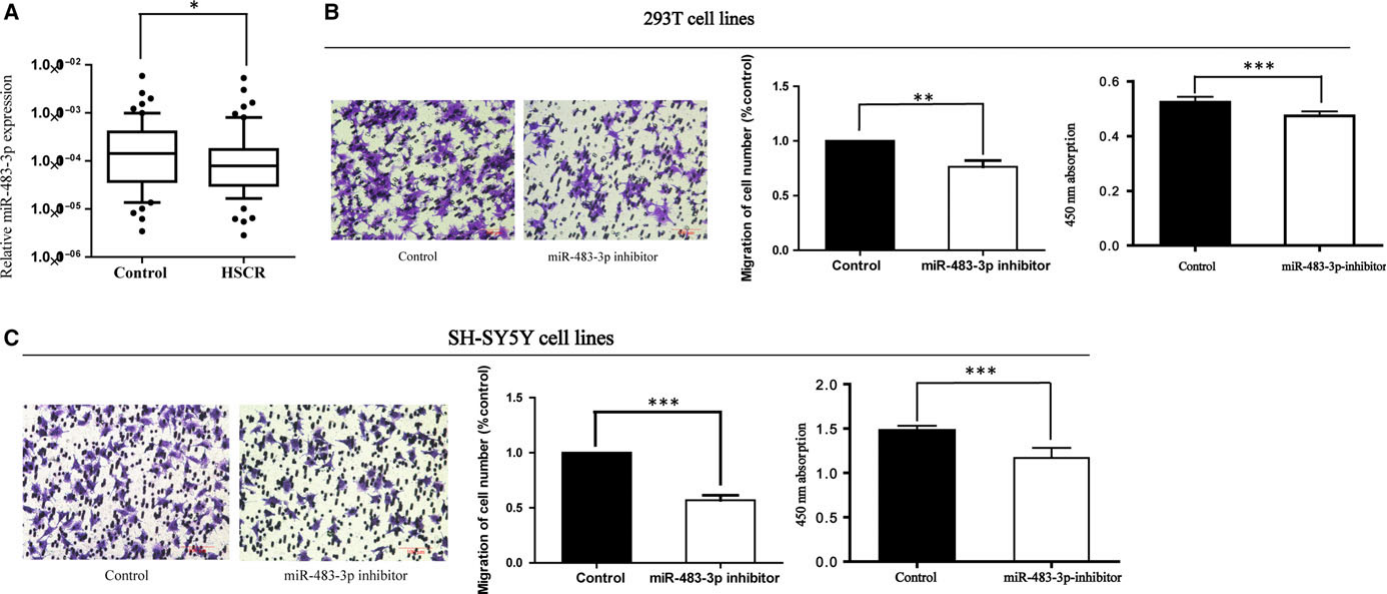
Down‐regulation of miR‐483‐3p in HSCR patients. A, The expression of miR‐483‐3p was detected in HSCR patient colon tissues (n = 60) and controls (n = 60). Data are presented as a box plot of the median and range of log‐transformed relative expression level. (*P < 0.05) B,C, The cellular function of miR‐483‐3p was verified in 293T and SH‐SY5Y cells transfected with miR‐483‐3p inhibitor. Migrated cells were then coloured with violet crystal and counted. The relative quantity of migrated cells is presented as a percentage of total cells. (**P < 0.05; ***P < 0.05) Absorbance at 450 nm is presented as mean ± SE (***P < 0.05; ***P < 0.05)

MiR‐483‐3p was examined down‐regulated in HSCR cases in comparison with matched controls from our previous microarray of miRNAs. To validate the relative expression level of miR‐483‐3p in HSCR, qRT‐PCR was conducted. As the results showed (Figure 1A), miR‐483‐3p was indeed at a lower level in HSCR tissues (without ganglion cells) than in normal tissues. Therefore, it led us to the hypothesis that miR‐483‐3p probably takes part in the development of HSCR.

### 3.3 Reduction of miR‐483‐3p depressed cell proliferation and migration

To verify our conjecture, the functional roles of miR‐483‐3p were investigated via transfecting 293T and SH‐SY5Y cell lines with miR‐483‐3p inhibitor in vitro. As expected, cells transfected with miR‐483‐3p inhibitor underwent impairment of proliferation and migration (Figure 1B,C), indicating that the deletion of miR‐483‐3p had a negative effect on these cell functions. Meanwhile, except migration and proliferation, whether miR‐483‐3p could influence cell cycle and apoptosis was also determined. Different from the results above, although cells were transfected with miR‐483‐3p inhibitor, there was still no effect on cell apoptosis and cycle process checked by flow cytometry analysis (Figure S1).

### 3.4 IGF2, the host gene of miR‐483‐3p

**Figure 2:**
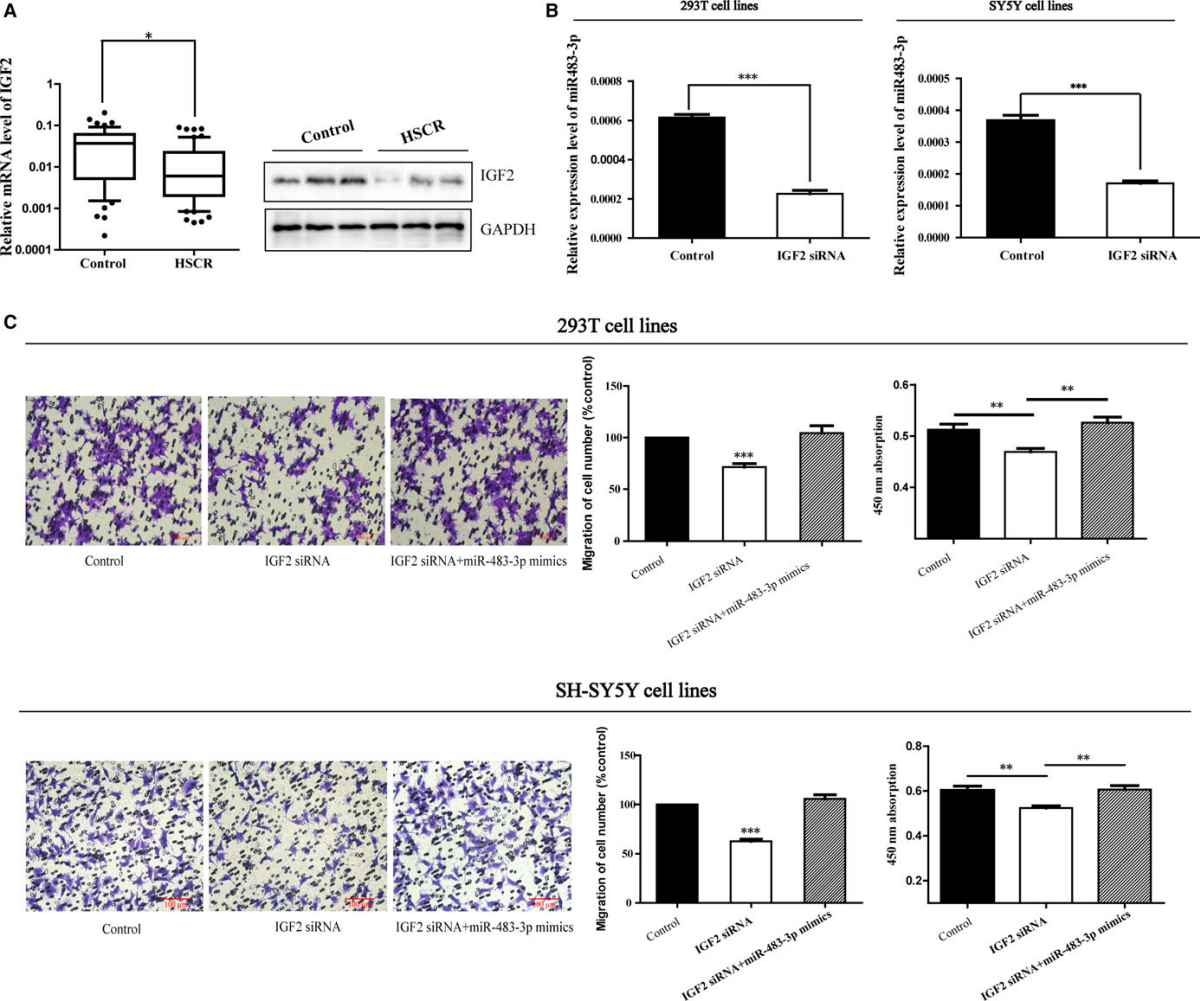
IGF2 is the host gene of miR‐483‐3p. A,The mRNA and protein expression levels of IGF2 were lower in HSCR tissues than controls determined by qRT‐PCR and Western blot. (*P < 0.05) B, The 293T and SH‐SY5Y cells were transfected with IGF2 siRNA, and then, the expression of miR‐483‐3p was reduced in both cell lines. (***P < 0.05) C, Co‐transfection of miR‐483‐3p mimics partially rescued the IGF2 siRNA‐mediated decrease in cell migration (***P < 0.05) and proliferation (**P < 0.05). Absorbance at 450 nm was presented as mean ± SE. *means significant difference (*P* < .05)

By bioinformatics analysis, we found that miR‐483‐3p resides in the intron 2 of its host gene IGF2 (at chromosome 11p15.5). On account of their relationship, the expression level of IGF2 in HSCR cases was also assessed. Results revealed that the protein and mRNA expression levels of IGF2 were both lower in HSCR than controls (Figure 2A). Whether the down‐regulated IGF2 caused the parallel reduction in miR‐483‐3p, the regulation mechanism between IGF2 and miR‐483‐3p was elucidated in the next step. IGF2 siRNA was transfected into 293T and SH‐SY5Y cells to weaken IGF2 expression, and then, the expression of miR‐483‐3p was repressed obviously at the same time (Figure 2B) in both cell lines. Therefore, IGF2 served as the host gene of miR‐483‐3p and regulated the expression of miR‐483‐3p.

### 3.5 The influence of IGF2 on cell migration and proliferation

Considering the down‐regulation of IGF2 in HSCR and its correlation with miR‐483‐3p, we suspected that IGF2 might take part in the pathogenesis of HSCR to a certain extent mediated by miR‐483‐3p. The following experiments where cells were transfected with IGF2 siRNA or along with miR‐483‐3p mimics have been performed for proving our hypothesis. As the results of Transwell and CCK8 assays showed, not only migratory but also proliferative cells were reduced markedly based on the low expression of IGF2 (Figure 2C) and just like what we have assumed, miR‐483‐3p mimics reversed the function of IGF2 siRNA (Figure 2C)***.*** Besides, whether IGF2 could impact the cell cycle or cell apoptosis was also tested, although there was no influence on them (Figure S1).

### 3.6 FHL1 is a direct target gene of miR‐483‐3p and up‐regulated in HSCR

**Figure 3:**
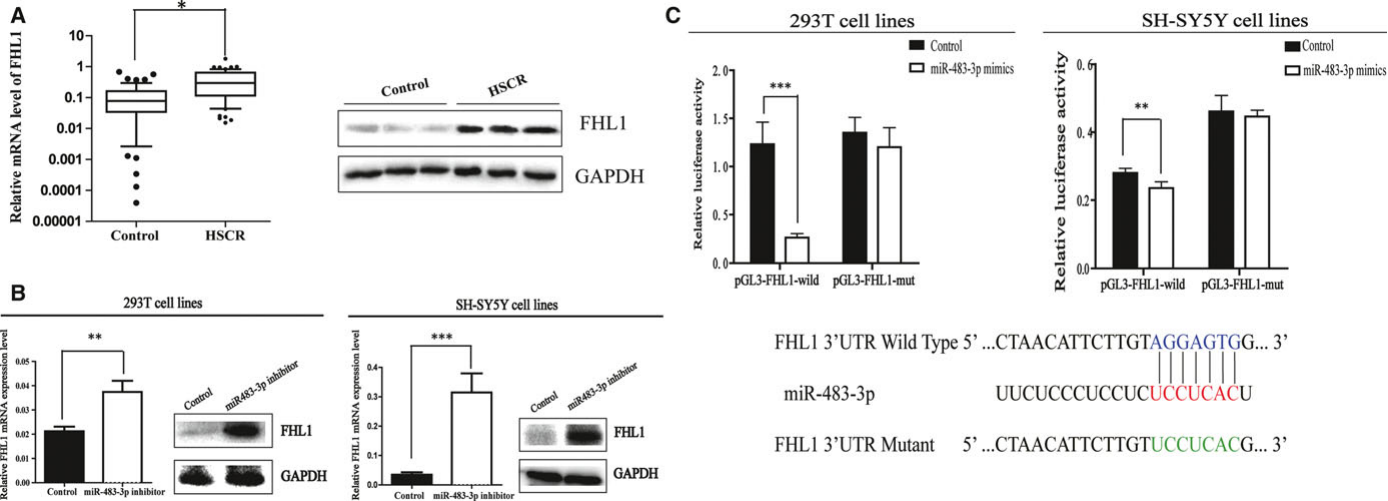
FHL1 is a downstream target of miR‐483‐3p. A, FHL1 was found up‐regulated in HSCR patients at both mRNA and protein levels. (*P < 0.05) B, The mRNA and protein expression of FHL1 were determined by qRT‐PCR and Western blot after transfected with miR‐483‐inhibitor in 293T and SH‐SY5Y cells. (**P < 0.05) C, Bottom: Sequence alignment of human miR‐483‐3p with 3′‐UTR of FHL1. Mutations in the 3′‐UTR of FHL1. TOP: The firefly luciferase activity in 293T and SH‐SY5Y cells after co‐transfection with reporter construct and miR‐483‐3p mimics (***P < 0.05; **P < 0.05)

To our knowledge, miRNAs often take part in the pathological progress through its downstream genes, so bioinformatic prediction was applied to clarify the probable target genes of miR‐483‐3p in this study. After the selection and analysis, FHL1 was chosen to be the potential gene of interest. Firstly, the relative expression of FHL1 in HSCR was checked by qRT‐PCR and Western blot, respectively. As indicated by the results (Figure 3A), the expression levels of mRNA and protein were up‐regulated compared with controls. Secondly, to further assess the possible regulatory relationship between miR483‐3p and FHL1, we transfected 293T and SH‐SY5Y cells with miR483‐3p inhibitor and negative control. FHL1 was found overexpressed at mRNA level as well as protein level in both cell types after transfected with miR483‐3p inhibitor for 24‐48 hours (Figure 3B). Finally, the wild‐ and mutant‐type luciferase plasmids were constructed for the Dual‐luciferase reporter assay. In the subsequent experiment, miR‐483‐3p decreased the luciferase activity of the wild‐type pGL3‐FHL1, but not the mutant‐type pGL3‐FHL1‐mut (Figure 3C). In combination with the above results, we demonstrated that FHL1 is a direct target gene of miR‐483‐3p and up‐regulated in HSCR.

### 3.7 Knockdown of FHL1 partially rescued the function of miR‐483‐3p inhibitor

**Figure 4:**
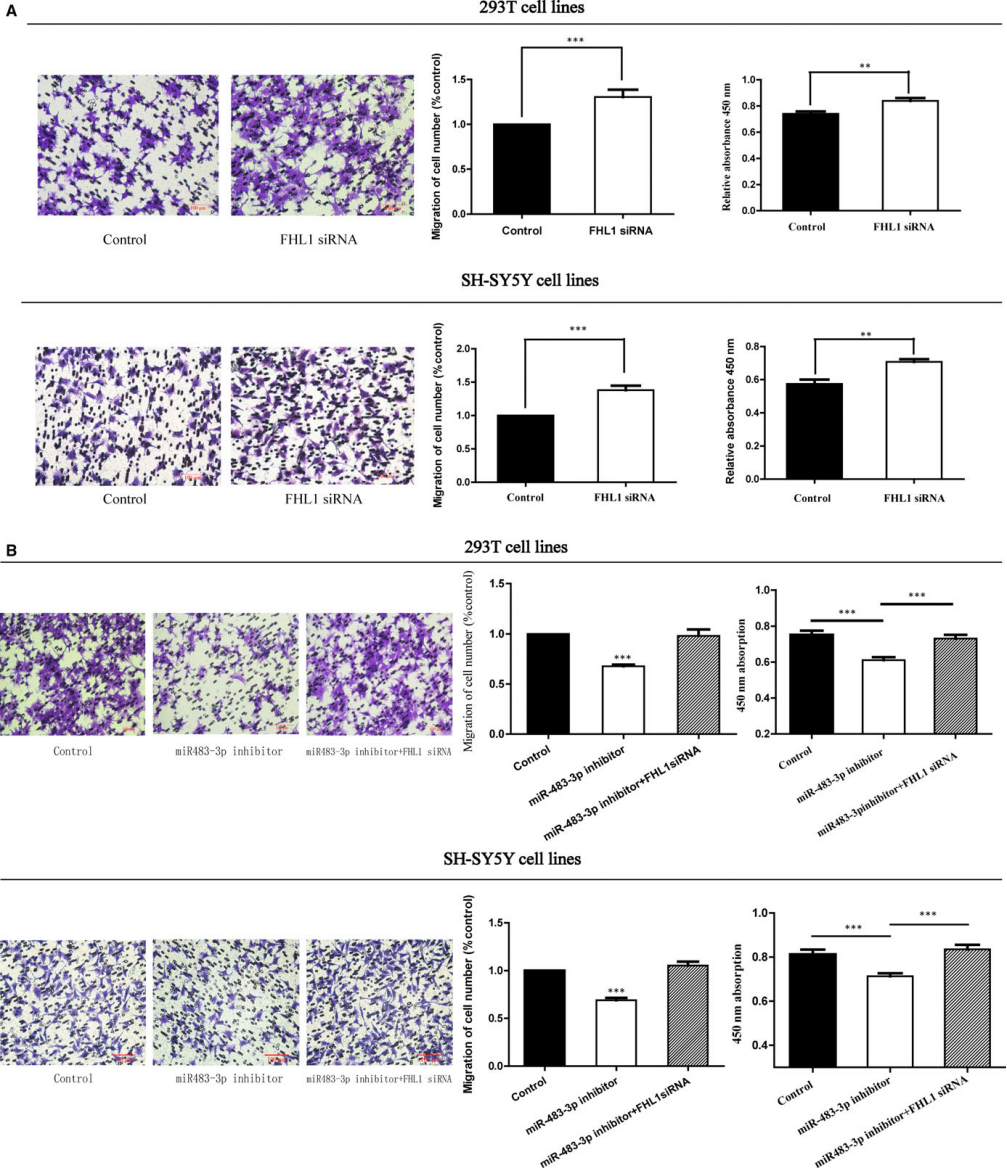
miR‐483‐3p effect on cell migration and proliferation was mediated by FHL1 in HSCR. A, The cell migration (***P < 0.05) and proliferation (**P < 0.05) in 293T/SH‐SY5Y cell lines transfected with FHL1 siRNA were improved than controls. B, FHL1 siRNA could partly reverse cell migration (***P < 0.05) and proliferation (***P < 0.05) in 293T and SH‐SY5Y cell lines transfected with miR‐483‐3p inhibitor. Absorbance at 450 nm was presented as mean ± SE. *means significant difference (*P* < .05). All results were presented as mean ± SE. *means significant difference (*P* < .05)

Owing to the overexpression of FHL1 in HSCR cases, elucidating whether FHL1 played a role in the progress of HSCR or not was urged; thus, a series of rescue experiments were designed in vitro to make it clear. Herein, we specifically used siRNA to depress the expression of FHL1. In both cell lines, after transfected with the FHL1 siRNA, the migration and proliferation capacities of cells were raised by the FHL1 siRNA compared with the negative control (Figure 4A). Interestingly, a phenomenon was discovered that the loss of FHL1 could partially rescue the suppressed action of miR‐483‐3p inhibitor on cell migration and proliferation (Figure 4B). The potential function of FHL1 on cell cycle and apoptosis was also detected here, but no difference was observed between the experimental group and control group (Figure S1). Ultimately, these results indicated that the abnormal expression of miR‐483‐3p impaired the function of cell migration and proliferation via keeping the higher expression level of FHL1.

## 4 DISCUSSION

Surgery is still the most important therapeutic method for infants who are suffering from HSCR and the definitive diagnosis of HSCR depends on histological identification of intestinal wall biopsy lesions during surgery.^27^, ^28^Unfortunately, despite children with HSCR have accepted surgery treatment, they are often probably accompanied with bad long‐term outcomes (eg, constipation) or even Hirschsprung‐associated enterocolitis (HAEC).^29^ Many researches suggested that coding genes were related to HSCR mainly including RET, GDNF, EDNRB and SOX10.^30^, ^31^ Based on our previous studies, besides these conventional coding genes, noncoding RNAs comprise miRNAs and lncRNAs also could take part in the development of HSCR.^32^, ^33^, ^34^ Different from traditional therapies, gene therapies will be more accurate and effective. It enhances us to understand HSCR from genes in order to create alternative therapeutic approaches to reduce the pain of children who have got HSCR.

MiR‐483‐3p derived from its host gene IGF2 as well as miR‐483–5p. The functions of miR‐483‐5p have been studied a lot in recent years. In adrenocortical cancer, miR‐483‐5p promoted tumour aggressiveness by regulating the expression of N‐myc downstream‐regulated gene family members 2 (NDRG2).^35^ In lung adenocarcinoma, miR‐483‐5p was regarded as a critical β‐catenin‐activated prometastatic miRNA and enhanced invasion and metastasis of cancer via targeting Rho GDP dissociation inhibitor alpha (RhoGDI1).^36^ In another research, the ectopic expression of miR‐483‐5p was correlated with tumorigenesis.^37^ Interestingly, this study also demonstrated that miR‐483‐5p could promote IGF2 transcription in turn by enhancing the RNA helicase DHX9 associated with the IGF2 transcript. For miR‐483‐3p, it has been reported dysregulated in multiple cancers, implying it could participate in regulating cell functions, such as growth and apoptosis.^38^, ^39^

IGF2 expresses only from the paternal allele and displays genomic imprinting (which is regarded as an epigenetic control) in common situation.^40^ So, except the roles of its intronic miRNAs, a number of studies focused on the epigenetic alterations of IGF2 which called LOI (the reactivation of the silenced allele of an imprinted gene is loss of imprinting.). LOI of IGF2 may be involved in the development of cancers because of its effects on cell proliferation and migration. Therefore, in diverse human cancers, like Wilm's tumour^41^and prostate cancer,^42^ the increase in IGF2 caused by LOI has been studied a lot.^43^ However, the abnormal expression of miR‐483‐3p and IGF2 has not been reported in HSCR. Different from those previous studies, we found the expression of IGF2 was lower in HSCR than controls, so the epigenetic changes (LOI) of IGF2 may not occur in the development of HSCR. It made us to explore its underlying mechanism in HSCR.

Here, as our data revealed that miR‐483‐3p and IGF2 were both down‐regulated in HSCR tissues compared with normal tissues. To further confirm the roles of them in the pathogenesis of HSCR, we transfected 293T and SH‐SY5Y cell lines with miR‐483‐3p inhibitor and IGF2 siRNA, respectively, and then, the cell migration and proliferation were impaired in both cell lines when compared to the negative controls. Owing to IGF2 is the host gene of miR‐483‐3p, we reached a hypothesis that the function of IGF2 maybe mediated by miR‐483‐3p in HSCR. During the next step, cells were transfected with IGF2 siRNA and miR‐483‐3p mimics collectively. In contrast to the former result, the function of IGF2 siRNA on cell migration and proliferation was reversed partly by miR‐483‐3p mimics. Furthermore, these results indicated that the miR‐483‐3p might play a greater role than IGF2 in HSCR.

FHL1, the target gene of miR‐483‐3p, has been confirmed by Dual‐luciferase reporter assay in this study. FHL1 has an elucidated vital role in the function of cell proliferation, differentiation and apoptosis^44^ and has been shown anomalous in various malignant tumours including breast, kidney, prostate^45^ and gastric cancers.^46^ On account of its important roles in the neoplasm, we were eager to investigate the underlying mechanism of FHL1 in HSCR when both the mRNA and protein expressions were identified at a higher level in aganglionic intestinal segments by qRT‐PCR and Western blot. After we transfected FHL1 siRNA into 293T and SH‐SY5Y cell lines for about 24‐48 hours, the number of both migratory and proliferative cells was increased compared with the negative controls. Then, cells were co‐transfected with miR‐483‐3p inhibitor and FHL1 siRNA to see whether the function of miR‐483‐3p was mediated by FHL1 or not. As supposed, the results suggested that the negative effects of miR‐483‐3p inhibitor on cell migration and proliferation were rescued by FHL1 siRNA obviously. Besides, the influences of IGF2, miR‐483‐3p and FHL1 on cell cycle and apoptosis were also estimated; however, there was no difference observed in this study.

Taken together, this study revealed that miR‐483‐3p transcribed from IGF2 may have great contributions to the pathology of HSCR by regulating the expression of FHL1. Therefore, our study provided a new approach for understanding the pathogenesis of HSCR and might contribute to a novel approach to the therapy of HSCR in future.

Nevertheless, there were still some insufficiencies in this study owing to the lack of animal model and the difficulty of using enteric neural crest cells to perform cell function experiments. Hence, more in‐depth studies are needed in future.

## CONFLICT OF INTEREST

The authors confirm that there are no conflicts of interests.

## AUTHORS’ CONTRIBUTION

Weibing Tang, Hongxing Li, Zhengke Zhi conceived and designed the experiments; Zhengke Zhi, Hairong Zhu, Xiaofeng Lv, Changgui Lu, Yang Li, Feng Wu, Lingling Zhou performed the experiments; Zhengke Zhi analysed the data and wrote the manuscript. All authors discussed the results and commented on the manuscript.
