# Corrigendum

**DOI:** 10.1111/jcmm.14142

**Published:** 2019-01-28

In Zhi et al,^1^ the original article contains errors in Figure 2C. The correct figure is shown below. The authors confirm all results and conclusions of this article remain unchanged.

The correct figure has been updated in the online version.

**Figure 2:**
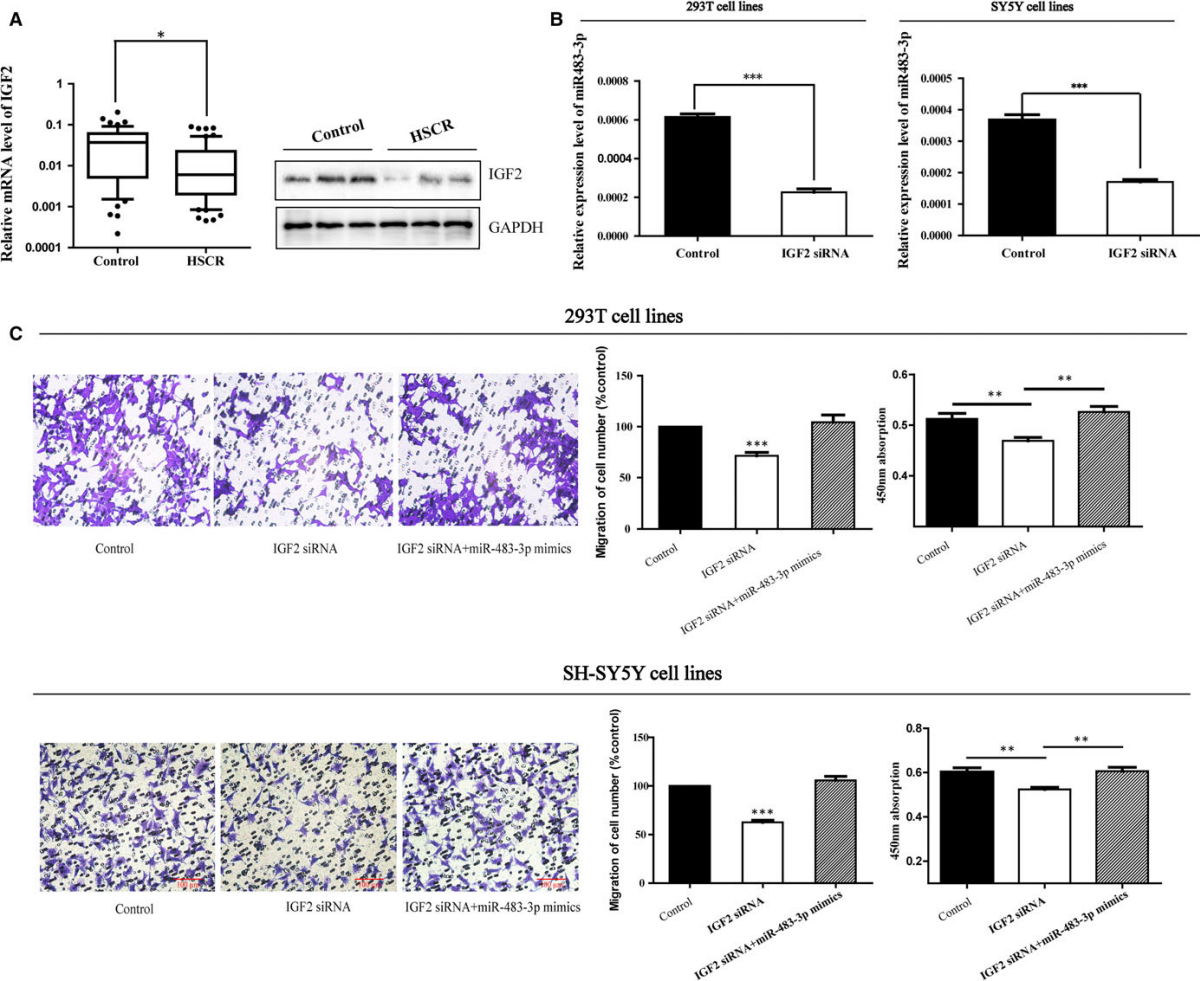
IGF2 is the host gene of miR‐483‐3p. A, The mRNA and protein expression levels of IGF2 were lower in HSCR tissues than controls determined by qRT‐PCR and Western blot. B, The 293T and SH‐SY5Y cells were transfected with IGF2 siRNA and then the expression of miR‐483‐3p was reduced in both cell lines. C, Cotransfection of miR‐483‐3p mimics partially rescued the IGF2 siRNA‐mediated decrease in cell migration and proliferation. Absorbance at 450 nm was presented as mean ± SE. *means significant difference *P < 0.05, **P < 0.01, ***P < 0.0001

The authors wished to apologize for any misunderstanding or inconvenience this may have caused.
