# Supplementary figures and images for: IGF2‐derived miR‐483‐3p associated with Hirschsprung's disease by targeting FHL1
Source: J Cell Mol Med. 2018 Aug 2;22(10):4913–21. doi: 10.1111/jcmm.13756 (PMC6156468; doi:10.1111/jcmm.13756)

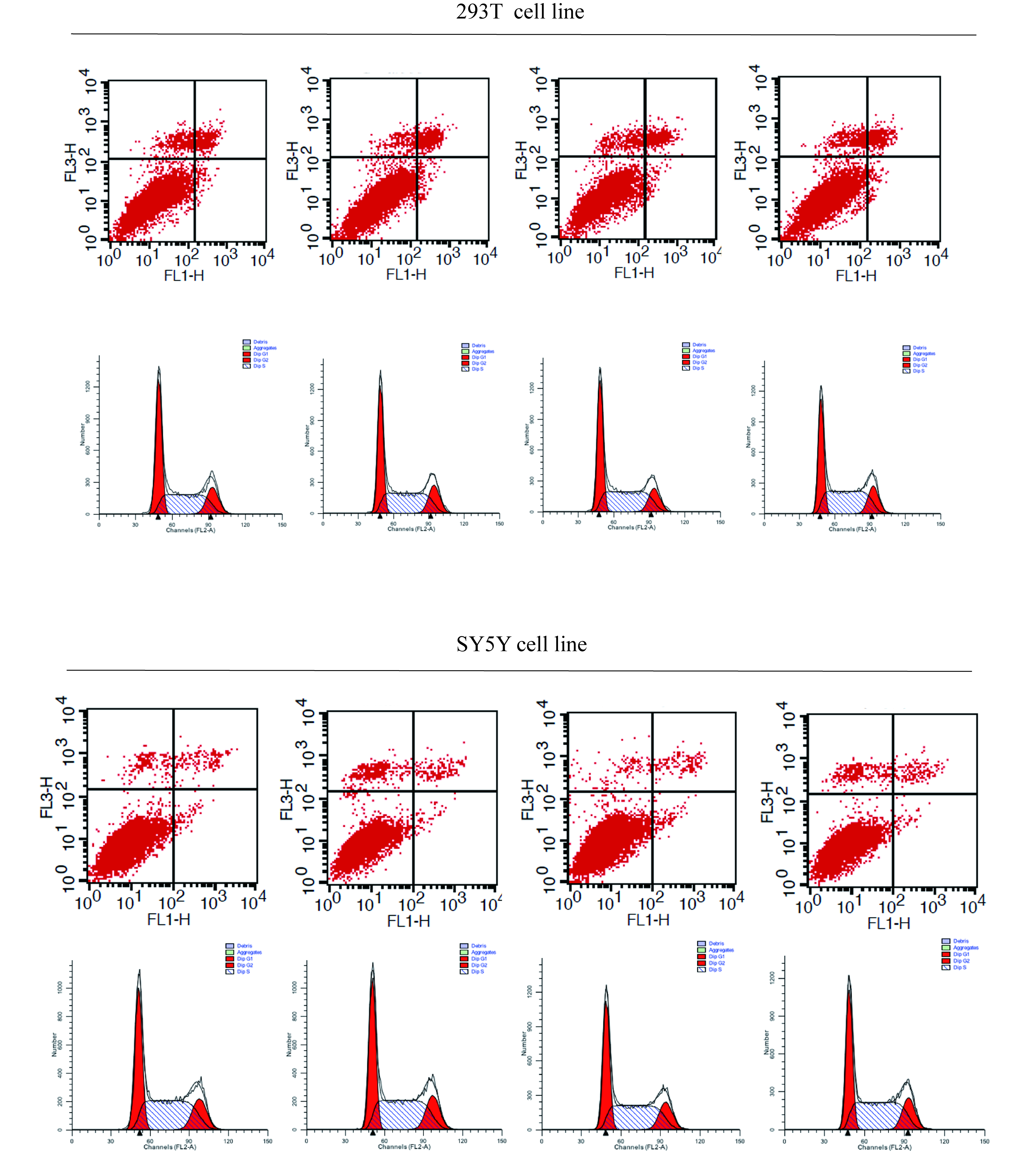

Supplement: Supplementary file 1 [file JCMM-22-4913-s001.tif]
